# Parent's Perception regarding the Delivery of Sexual and Reproductive Health (SRH) Education in Secondary Schools in Fiji: A Qualitative Study

**DOI:** 10.1155/2020/3675684

**Published:** 2020-01-09

**Authors:** Sharan Ram, Sari Andajani, Masoud Mohammadnezhad

**Affiliations:** ^1^School of Public Health and Primary Care, Department of Public Health, Fiji National University, Suva, Fiji; ^2^School of Public Health and Psychosocial Studies, Department of Public Health, Auckland University of Technology, Auckland, New Zealand

## Abstract

**Background:**

Adolescent Sexual and Reproductive Health (SRH) remains a challenge globally. This study aims to gauge the perceptions of parents towards the delivery of SRH education in mainstream public secondary schools in Fiji.

**Methods:**

The qualitative study design was used to collect the data from parents in Suva, Fiji, from July to August 2018. A semistructured questionnaire was developed to run Focus Group Discussion (FGD) among parents residing in Suva who had school-attending children from years 11 to 13. Parents were recruited from five schools with the help of students. Twenty-six parents of which 10 were males, aged between 38 and 65, participated in this study. Consent was obtained from each participant prior to the data collection stage. Data collected were transcribed verbatim and were analyzed thematically. Ethical approvals were obtained before collecting the data.

**Results:**

Seven themes emerged which included the provision of school-based sex education, parental involvement with school-based sex education, sex education at home, age-appropriate incremental sex education, ethnic variations regarding sex education, barriers and facilitators for the delivery of school-based sex education, and perceived ideal version of sex education.

**Conclusions:**

Findings from this study suggest for policy and programs to match parents, schools, and students' expectations. Effective interventions need to involve and help parents to take a more active part to change policy, program, and advocacy for relevant SRH education.

## 1. Introduction

Sexual and reproductive health (SRH) is an important public health issue globally [1–3]. SRH is defined as a state of complete physical, mental, and social well being in all matters relating to the reproductive system [4]. SRH recognizes the rights of people to have a satisfying and safe sex life and the freedom to decide if, when, and how often to do so [5, 6]. Adolescence is an opportune time to build healthy habits and lifestyles relating to SRH. Adolescence is a critical development period, marked by the years between the onset of puberty and the establishment of social independence [7]. Key SRH issues that affect young people are puberty, pregnancy, access to modern contraceptives, unsafe abortions, and violence including gender-based violence [8]. In developing countries, 11 percent of females and 6 percent of males aged 15–19 have had first sex before the age of 15. Significant gender disparities exist in this area: while adolescent boys were more frequently engaged in higher-risk sex, they were also more likely to use condoms than adolescent girls; compared to boys, adolescent girls are more likely to experience the physical and emotional burden of unintended pregnancies and childbirth-related complication [9, 10]. Additionally, gender-related protection risks such as sexual and gender-based violence in many countries and communities make it especially important that adolescent girls are empowered with requisite SRH knowledge and access [11, 12]. In Asia and the Pacific region, 33 of every 1000 births are among adolescents between the ages of 15 and 19 [13, 14].

The adolescent population in Fiji comprises more than 25% of the total population which is 884,887 [15]. According to the Fiji Ministry of Health (MoH, 2013), the teenage pregnancy rate in Fiji has increased significantly in the past five years. There was an increase of 95%, in the year 2013 compared to the previous year. The rate of teenage pregnancy increased by sixfold within a year, from 4.91 in 2014 to 24.3 in 2015. This was particularly alarming and was attributed to improved reporting. In Fiji, the human immunodeficiency virus (HIV) incidence has increased over the last 25 years from 0.7 to 7 per 100,000 populations [16].

The role of the education system in sexual health promotion has been widely acknowledged [7, 17]. There is a plethora of evidence showing that adolescents who receive HIV and sex education are less likely to engage in sexual activity and more likely to engage in safer sexual practice [18]. To stay healthy and safe, adolescents need access to high-quality and relevant SRH services and information from an early age [19, 20].

Ample studies show how Comprehensive Sexuality Education (CSE) can play a central role in the preparation of young people for a safe, productive, and fulfilling life [8, 21]. A study of high school students in Chicago showed that students who had never received any CSE were more likely to engage in high-risk sexual behaviors than their peers in schools with CSE [22]. Students who received SRH education were less likely to experience teenage pregnancy, engage in unprotected sex, and have multiple sexual partners [23]. They also tend to delay sexual initiation and use a condom or other methods of contraception [24]. Studies also confirmed parents' positive attitudes toward CSE such as those in Canada, the Netherlands, and some states of the USA. Studies show that in some countries like Canada, parents have a favourable attitude towards the importance of school sex education. Parents want sexual health education and its content to be age-relevant, for example, whether it focuses on abstinence-only or abstinence-plus based on the adolescent's mental maturity [25, 26].

In Fiji, reluctance and hesitance about breaking perceived taboos related to adolescent sexuality and addressing teenage pregnancies directly and openly are commonly mentioned by parents. Adolescent reproductive health in Fiji is continuously a sensitive and controversial topic [27]. SRH education was first introduced in 1985 and resurrected in 2007–2008 due to concerns over teenage pregnancy and STIs in adolescents [21].

Unfortunately, despite instituting a sex education program in schools, unintended teenage pregnancy, STIs, and HIV continue to increase. This study explored parents' perception and aspiration towards sex education in mainstream public secondary schools in Fiji as well as parents' understanding of important topics in SRH and barriers to the delivery of SRH in high school.

## 2. Methods

### 2.1. Study Design and Subjects

This study utilized a qualitative research method to collect data using focus group discussions (FGDs) among parents who had high-school attending children in Suva, Fiji, from July to August 2018. The target population was parents residing in Suva who had school-attending children from years 11 to 13 (i.e., 15-18-year-olds), irrespective of gender, in mainstream public secondary schools in Suva, Fiji. The study excludes any parent who did not have high-school attending children.

### 2.2. Sample Recruitment

A list of secondary schools was obtained from MOH, and schools with equal ethnic mix were selected to ensure equal representation from the two ethnicities, which are iTaukei and Indo-Fijian. To recruit the parents, SRH classes were visited and an oral presentation on the study rationale was made. Students were provided blank pieces of paper. At the end of the presentation, students were asked to indicate if their parents would be available for interviews and if so to write their name and contact address. Parents were then contacted by phone to verify their availability, explained about study rationale, and verbal consent was obtained for participation in the study. A convenient time to convene for FGD was discussed via phone.

### 2.3. FGD Procedure

The focus groups were made up of at least a minimum of 4 individuals. Five focus group discussion sessions were held and the duration of each session was up to 40 minutes. FGD with male parents was conducted in the evenings while for female parents, FGD was conducted during the daytime. Issues about SRH are sensitive, and thus, the privacy of the respondents was given serious consideration. FGD amongst the male parents was facilitated by the researcher (male) himself, while FGD with the female parents was led by a female research assistant who was a bilingual *iTaukei* and trained on how to facilitate FGD and FGD process. Separate FGDs were conducted based on the gender of the parents. This was to encourage openness and allow for gender-specific and gender-sensitive responses from the participants. This also helped to ensure a flow of individual response and group discussion as a gendered subcategory, perhaps sharing similar experiences. The questions formulation was piloted to ensure the cultural relevance and sensitivity of the questions asked. The FGD facilitators were both born and raised in Fiji and were familiar with the relevant cultural norms and social mores and gestures appropriate in Fiji culture. Participants were reminded that their participation was voluntary and information was to be treated confidentially. The FGD participants were reminded of the importance of respecting opinions, views, and beliefs of each participant and that there were no right or wrong answers.

The FGDs were conducted by the facilitator and, with participants' consent, were digitally recorded. Prior to each FGD, participants were given the study information sheet explaining the study rationale and later, once again, participants were explained the study rationale in vernacular. Once they agreed to participate, they were required to sign the consent forms. All individuals' participation was voluntary.

The researcher strived to ensure that the place used for FGD was quiet and cool and provide a sense of privacy for the participants. FGD was terminated once there no new information was gathered.

### 2.4. Data Collection Tool

To gather qualitative data, a semistructured open-ended questionnaire was used to guide the FGD. The questions were adapted from a similar study conducted by UNESCO in four Pacific Island countries in 2015 [28–30], which was an attitudinal survey conducted amongst principals, teachers, parents, and students. The FGD guides were piloted in Fiji and as such it is assumed to be a validated tool.

Trustworthiness was incorporated in this study by using Lincoln and Guba's (1985) strategies of creditability, transferability, dependability, and conformability [31].

### 2.5. Data Analysis

Audio-recorded FGDs were first transcribed into English verbatim. Only one recording needed to be translated from iTaukei to English and then transcribed. The transcribed data were read and re-read, and the audio recordings were listened to multiple times to immerse in the data. In doing so, the key categories/themes were identified, and data were summarized under appropriate categories [32].

### 2.6. Ethical Approval

Ethical approval was sought and granted by the Fiji National Universities' College Health Research & Ethics Committee (CHREC); the Fiji National Research Ethics Committee (FNREC) of the Ministry of Health; and the Fiji Ministry of Education, Fiji. Due consent was obtained from the participants regarding the audio recording of FGD, and participants were assured anonymity.

## 3. Results

Five FGDs with parents were convened within the Suva and Nausori area in Fiji. Twenty-six parents (10 males) participated whose ages ranged from 38 to 65 years old (Table 1).

Seven themes emerged which are the subheadings including the voices of the parents are also quoted.

### 3.1. Provision of SRH Education in Schools

Across all groups, all parents were in favour of the provision of SRH education in schools and said that it was crucial to have sex education in schools. Parents mentioned that issues around SRH were not discussed openly in the majority of the homes as it is still considered a taboo, and thus, they felt that the schools were a good avenue to teach young people SRH.

Parents thought that it was important that they start teaching on avoiding courtship in high schools, teenage pregnancy, and its effects, etcetera, particularly to the girls at a younger age as they are more vulnerable to unintended teenage pregnancy compared to the boys, as girls would be the one who would be getting pregnant. Young pregnant girls are condemned by society, and they are seen to bring shame and dishonour to the family. One male parent said

*“Parents should have “family talk” with their children to explain bad behaviors and negative consequences. This should also be reinforced at the school.”* (Indo-Fijian male parent)

On provision of sex education in school, another male parent stated that

*“I believe kids these days mature early, particularly due to their food habits and with maturity, there is a tendency of some to sleep together as well and we cannot stop that. I strongly suggest the education department provide sex education around Year 7/8.”* (iTaukei male parent) (see quote 3.1.1 in Supplementary materials).

### 3.2. Parental Involvement in SRH-Delivered Schools

Some parents knew that there were sex education classes in schools and the majority knew that some sexuality and sexual health-related information was taught in science classes.

None of the parents who participated in the study was ever consulted concerning the delivery of SRH education in schools; however, all parents mentioned that they would be keen to be consulted. Some parents felt that they were not educated nor trained and prepared to talk about SRH issues with their children at home. Parents were keen to know what (content) was being taught in schools and how they could reinforce what their children learned in schools at home. Parents were eager to take an active role and participate in SRH education for their teenage children. Parents mentioned that teachers-parents day was the only time they visited the school to talk with their children's teachers and the discussion was mostly on the children's academic progress as one parent shared the following.

When parents were asked, “*Would you like if teachers invited you to school to discuss what they teach on sex education school?”* Indo-Fijian male participants said

“*I think it is very important for us parents to know what our children learn at school. On parent's day whatever they tell, we just listen mostly on academic stuff. It will be good if this subject is also discussed.”*

The majority of the parents mentioned that they had no knowledge of which subject was related to SRH or the content of SRH taught in the school.

### 3.3. SRH Education at Home

The majority of the parents mentioned that they did not talk about SRH issues at home because it is considered taboo in both iTaukei and Indo-Fijian culture. Parents only limited themselves to telling children not to engage in courtship particularly when they are still in high schools and that their focus needs to be education. They had never discussed with their teenage children on either contraception or prevention of STI/HIV. Parents had assumed that concepts related to reproduction and contraception were being taught in schools; however, they were not sure about it and had no idea what the content was. A father said

*“I have never in my life talked to my children on these (SRH). I think [guessed] my wife would be talking to my daughters on these issues”* (Indo-Fijian male parent).

Parents felt that they have some responsibility for providing SRH information to their teenage children and that teachers could also share this responsibility (see quote 3.3.1 in Supplementary materials).

Parents mentioned that the sensitive topics that are not taught at home would be better discussed in schools. However, parents felt that this may require greater communication between parents and the school team (see quotes 3.3.2 and 3.3.3 in Supplementary materials).

Parents also agreed to be aware of the danger of STIs, but they were not talking to their teenagers about it and would expect the schools to include such topics in the school subjects.

Parents strongly felt that SRH should be taught at the appropriate age levels. Parents also mentioned that SRH should begin early, especially for the girl child, when she just entered a primary school (around 6 years old). Despite feeling apprehensive and not well equipped to teach or discuss SRH matters with their children at home, parents believed that all parents, schools, and religious institutions should play their part and deliver appropriate knowledge from their perspective.

One parent mentioned that SRH for a female child is supposed to be slightly different from the male child because females are more vulnerable in terms of ending up with teenage pregnancy (see quote 3.3.4 in Supplementary materials).

There was agreement on this by the rest of the participants of the male parent group.

### 3.4. Age-Appropriate Incremental Sex Education

There was a consensus in all groups that sex education content should be incremental. Everyone agreed that it should begin at the primary school level with some general health education and that as the students move up the levels, the content should be more in-depth (age-relevant) and gender-sensitive. One guardian mentioned that at the primary school level children should be taught about hygiene and what is “good touch” and “bad touch”—whereby a good touch is when, for instance, an adult pats a child on the back for good behavior making them feel happy while a bad touch is when someone touches their private parts. She added that there must be ways teachers could teach sex education to kids which are different from the way older students are taught.

The elderly female guardian stated

*“My 5 years old grandson has access to the internet and he accesses stuff on the internet we think he is ignorant about or not appropriate for his age. Thus I think education should start as early as from pre-school, maybe not the way we teach the older ones......just like a “bad touch” and a “good touch”. There has to be a way to teach sex education to kids and teachers should be trained on this. Some education at home is most important”* (female guardian, mixed ethnicity).

### 3.5. Ethnic Variations in Perceptions towards SRH Education

Very few ethnic variations were noted in the perceptions between the two main ethnicities. The one noted was between the Indo-Fijian female parents and iTaukei female's parents. For the Indo-Fijian mothers, it was noted that they mentioned that with the increase in rape cases, they were prepared to move out of their comfort zone and challenge their own beliefs about sexuality as taboo and willing to share as much as they could, particularly with their daughters, as they were more vulnerable (see quote 3.5.1 in Supplementary materials).

On the other hand, some of the iTaukei female parents claimed that their Fijian communal lifestyle encouraged meet-ups of teenagers in the evenings and at late nights with the potential for them to engage in sex. For iTaukeis, the houses are very close to each other and a lot of time the boys are on the outlook for the girls and they are awake till late. Sometimes, the females sneak out at night to see boys for courtship and potential sexual intercourse. Additionally, mothers found girls play truant and end-up with boyfriends. As such, some mothers mentioned that they would, without hesitation, accompany their daughters to a family planning clinic and get them to have an implant as keeping close supervision was proving difficult and they did not know when their adolescent females would sneak out and engage in sexual activities. One iTaukei mother shared

“*In our settlement, teenage pregnancy is a big issue and as moms, they should take their daughter for an implant even when they are in secondary school. I have a granddaughter. She is not schooling. Her mom took her to the family planning clinic and now she has the implant for 5 years.”*

When they were asked “*If they are still in high school, do you think they should have an implant?”* an iTaukei participant said

“*Yes!!! because it is hard to be behind them (girls) or supervising them every minute especially at night as they make excuses for meeting friends or sneak out. So the only solution is to take your daughter to the clinic and give them the implant for 5 years so you don't have to worry about her getting pregnant.”*

### 3.6. Barriers and Facilitators for the Delivery of SRH Education among Parents

During the course of the discussion, barriers and facilitators became apparent. For instance, one facilitating factor noted was that all parents gave full approval of the sex education to be taught as quoted below:

*“I think sex education is very important nowadays, we are getting very modernised so kids have all types of gadgets, all technology and they are getting all that information from the technology…why don't we start teaching this from the pre-school level and give them information very openly because finally, they will know what to do...so why don't we teach them from pre-school...give all the detail on what is wrong and what is right”* (Indo-Fijian female parent).

The key barriers identified were parent's lack of knowledge on ways to discuss sex education coupled with it being a taboo subject.

### 3.7. Ideal Version of SRH

Parents mentioned that when they or any elders told children not engage in sex, then children become more curious and they tried to do so. Thus, it is important to include morality/religious education to guide them too as one parent stated

*“I think sex education should be taught, however, we should include morality and religious education with it or children will start practicing what they are taught and pregnancies will go up”* (iTaukei male parent).

The Indo-Fijian male parents also agreed that there should be some teachings in religious groups and consistent messages coming from them so children pay heed to it.

One parent who has Y11 and Y5 school-going children said that it is important how teachers communicate/delivered sex education to kids. Teachers need to know the age appropriateness of what is delivered. He said he was not happy with how reproduction was taught to his child in Year 5 and felt that the topic was mishandled (see quote 3.7.1 in Supplementary materials). This is actually not a negative reaction from a parent, but a parent that would like to see an age-appropriate sex education. Teachers should start with basics at the younger level and teach on reproduction at the onset of puberty.

Parents felt that ideal sex education would be one where they are consulted, and all parties ensure the same consistent message was delivered to students.

Parents also felt that teachers need to be trained in delivering sex education. They (teachers) should know when to teach a certain concept and understand the dynamics of their class, such as the relationships and cultural aspects of Fijian culture that existed in the classroom. For instance, in the iTaukei culture, males cannot approach or talk to females on certain topics and this is called “veitabuki” (See quote 3.7.2 in Supplementary materials). The teachers who are communicating need to be equipped, mature, and have the wisdom to deliver such education.

## 4. Discussion

The purpose of this qualitative study was to gauge the perceptions of parents regarding the delivery of SRH education in mainstream public secondary schools in Fiji and the way in which parents could play an active role in its improvement. This section presents an interpretation of the findings of this study.

In this study, parents favoured school-based sex education. Parents approved of school-based sex education because they perceived they had limited content knowledge on the subject and did not know how nor have the skills to approach this socially perceived to be a taboo topic. This social taboo as a hinderance for sexual education has been found in other studies, like those in Asia, Africa, and other countries in the Western Pacific [29].

There were some families which had made an effort to discuss the topic with their children; however, it was limited to a reprimand to their children about abstinence and prevention of unintended pregnancies.

The study showed the willingness of parents to be actively involved in the sex education of their children in schools and to be approached and consulted by the school. Many participants in this study had become aware of the importance of SRH and particularly, in light of increasing teenage pregnancy, higher incidence of STIs and HIV/AIDS, increased vulnerability of children to sexual abuse and exposure of children to unsafe social media and technology. Participants were keen to reinforce whatever is taught in the school; at home environment, parents' involvement in children's sex education has a positive impact on their children's future sexual health and behavior [25].

This study highlights that it is very important for professionals/teachers to involve parents alongside other sources of sex education in health and educational strategies to address sexual health issues and improve sex education in Fiji. Parents have the interest to promote healthy sexual behavior of their children, which is in line with the national agenda on the sexual health and well being of young people and prevention of unwanted and teenage pregnancies and STI including HIV/AIDs.

Another study by Meschke and Peter concurs with the findings of this study on the important role of parents in supporting the implementation of a successful comprehensive sexual and reproductive health education [33].

Other reasons why sexuality and sex education are not discussed at home are because parents felt unknowledgeable on both the subject and how to talk about it with the children and they thought that teachers would be better trained for this. Participants in this study said that they were not trained nor did they know how to approach sex-related topics with their children, yet they advocated for comprehensive sexual education (CSE) and would like to be involved in which way relevant. Some participants strongly felt that discussion on contraception was very important, such as implants, to prevent teenage pregnancy even whilst their daughters were at school. In urban areas, participants acknowledged the difficulty to do surveillance over their child's movements and their whereabouts. Therefore, participants wanted their children to know different types of contraceptive methods and their side effects. A few parents did not oppose their teenage children to use contraception if needed as they did not want to see them (girls) getting pregnant out of the wedlock, while they were still at school and ruined their future.

These findings confirm other studies where caretakers (i.e., parents) and adolescent's communication on sex-related issues were very low and not comprehensive. The caretakers failed to communicate to their adolescent children sensitive topics such as on condoms and contraception and did so on less sensitive topics such as abstinence. Additionally, the pattern of communication varied across the genders of the caretakers/parents and that of the adolescents and depended on the nature of the relationship shared between the two [34–36].

This study reveals complexities in parental attitudes not reported elsewhere. For example, although parents believed that it was very important that their children be provided comprehensive sex education, they said in a school system, certain topics would make the situation awkward for the children due to existing complex cultural systems (i.e., values, beliefs, and norms around sexuality). As outlined in the results, parents suggested that separating the two genders for such sessions would be a way forward for sex education in schools and for the same gender teacher or facilitator for each female or male class.

This study showed that the majority of participants were hesitant, uncomfortable, and feeling not having the skills to discuss SRH-related matters at home. There was a consensus amongst all participants for SRH education to commence early, both at home and in schools; however, it must be noted that although parents have approved of SRH education, they have emphasized on age appropriateness of education. In the elementary grades, for instance, good hygiene education and “good and bad touches” could be taught and as the years progress by. more sensitive topics could be added, such as from the onset of puberty. This was because parents acknowledge an increase in teenage pregnancy, as well as rape, and it was felt that early education was needed to reduce the vulnerability of children, particularly females as discussed above. A review of reviews of SRH education has shown that age-appropriate comprehensive SRH education starts at primary schools [37].

iTaukei female parents differed from Indo-Fijian mothers in terms of what they could do to ensure the prevention of teenage unintended pregnancies. Some of the iTaukei mothers in this study felt that they could go to the extent of having their daughter get an implant done whilst Indo-Fijian mothers said they would ensure that they would get out of their comfort zone and be open in disseminating the SRH knowledge. Both of these groups resorting to these solutions can be explained by the fact that in both of these societies, mothers are seen as having a greater role and responsibility for the welfare of the girl child as opposed to the male parents. Mothers would have themselves to be socially blamed in an event unintended teenage pregnancy, especially if the girl is not married. This may concur with a study in which mothers of children with disabilities took a greater role compared to the father in providing sex education to protect children from sexual abuse [38].

Overall, the study described more barriers in the delivery of SRH education than facilitating factors. The key facilitating factor was the high level of parental support in favour of providing school-based sex education. As discussed earlier, this could be due to the inability of the parents to openly discuss SRH issues at home and considered schools as better avenues for their children to obtain sex education. Another reason for their strong approval was that participants believed that teachers are well trained to teach SRH while parents lack knowledge both on content and skills to teach SRH education at home. Unfortunately, this may not be the case, as other studies suggest the hesitation and lack of skills amongst teachers in SRH deliverance [39]. The barrier for parents was perceived as social taboo on any talk related to sexuality with children and lack of knowledge on SRH. Participants in this study acknowledged that they themselves had never received nor talked about sex with their parents and had never attended any SRH classes during their growing up times.

## 5. Strengths and Limitation of the Study

The study has several strengths. This is the first qualitative study in Fiji on the perception of parents on SRH education, and thus, it presents a new argument for SRH advocacy. The study reports findings from primary data. As such, the research data includes the views of parents of adolescent children. Additionally, this study can serve as a baseline to assess changes in parent's perception towards SRH education overtime, should there be a future mandatory implementation of sex education programs on a national level. In terms of the limitations, the two FGD were conducted in vernacular, and as such, there is a potential for loss of information during the translation process.

## 6. Conclusion

The study provided insights into the needs, as well as aspirations of the parents, regarding sex education in mainstream secondary schools in Fiji. Parents fully supported the delivery of CSE and preferred that it be started early, was age-appropriate, and be done in an incremental fashion. Acknowledging that SRH education is a taboo topic at home and that parents felt apprehensive, embarrassed, and ill-prepared to discuss SRH at home or rather discussion were limited to prevention of premarital sex and abstinence; it is important CSE age- and culture-appropriate SRH education be provided. Finally, parents suggest communication on the inclusion of relevant cultural norms, mores, beliefs, and practices to be included in the morality framework of SRH as well as respecting the gender-sensitive nature of the class setting.

## Figures and Tables

**Table 1:** Sample characteristics.

Focus group number	Gender and ethnicity	No. of participants
Focus group 1	*iTaukei* females only	5
Focus group 2	*iTaukei* males only	4
Focus group 3	Females of mixed ethnicity	7
Focus group 4	*iTaukei* females only	6
Focus group 5	Indo-Fijian males only	4
Total		26

## Data Availability

The data used to support the findings of this study are available from the corresponding author upon request.

## Supplementary Materials

Supplementary Materials Supplementary information includes the voices of the parents quoted under the appropriate themes. These are referenced in-text in the Results section.
