# Supplementary material for: Parent's Perception regarding the Delivery of Sexual and Reproductive Health (SRH) Education in Secondary Schools in Fiji: A Qualitative Study
Source: J Environ Public Health. 2020 Jan 9;2020:3675684. doi: 10.1155/2020/3675684 (PMC7201858; doi:10.1155/2020/3675684)
Supplement: Supplementary Materials — Supplementary information includes the voices of the parents quoted under the appropriate themes. These are referenced in-text in the Results section. [file 3675684.f1.doc]

# Appendix: Supplementary Quotations

The voices of the parents are quoted under the appropriate themes. These are referenced in-text in the Results sections.

## *3.1 Provision of SRH Education in Schools*

Quote:3.1.1

Another male parent said: *“From birth until age 6, kids are under close parental care and supervision, after that they spend more contact hours with teachers…..therefore majority of the time is spent with teachers and it would be good if they [teachers] provide sex education.”* [Indo-Fijian male parent]

## *3.3 SRH Education at Home*

Quote: 3.3.1

One male parent stated that: *“I think once students enter the school gate, then the students become the responsibility of the teachers and any unwanted action (courtship) should be corrected there and then.”*[Indo-Fijian male parent]

Quote: 3.3.2

One parent said: *“At home, every family should have “family talk” once in a while ....once a week maybe. Parents should ask about their child’s welfare and tell them if they engage in anything that they are not supposed to then it will have many consequences. If these things (sexual affairs) are reinforced in the schools as well this will have a good effect on them.”* [Indo-Fijian male parent]

Quote: 3.3.3

Another male parent added: “*I have never given any sex education to any of my kids. I think it is not only the job of the schools to provide sex education. I think we parents, schools, religious groups- all should be involved and give the kids the same consistent message. Once they find that they are getting the same message, they will believe it to be true and accept it*”. [Indo-Fijian Male parent]

Quote: 3.3.4

An Indo-Fijian meal parents He expressed that: *“SRH education for a male child is different from a female child. In our culture, females must maintain chastity until marriage or else it is considered bringing disrepute to the family if that, for instance, results in pregnancy which will make it all obvious. Thus, for girls particularly sex education should begin as early as 6-year-old at home and I think sex education is a must in schools.”* [Indo-Fijian male parent]

## *3.5 Ethnic Variations in Perceptions Towards SRH Education*

Quote: 3.5.1

Indo-Fijian mother states that: *“Nowadays with the increase in rape cases, and the blames being shifted to mothers, I have gathered the courage to be open to my children. I openly talk to them about issues and that they (kids) have to be careful. I also tell my son not to engage in any unwanted behaviour with the girls. He should also think that he has a sister too. I advise my daughter to dress appropriately.”* [Indo-Fijian female parent]

## *3.7 Ideal Version of SRH*

Quote: 3.7.1

One iTaukei parent stated that: *"One day my child of Year 5 came home and he said, “Daddy if I knew how I was born, I wouldn't want to be born". Clearly, he was disgusted by the thought of it and so teachers need to understand what needs to be taught at what level.”* [iTaukei male parent]

Quote: 3.7.2

One iTaukei male parent stated that: *“Some topics like sexual reproduction/pregnancy and contraceptive methods need to be taught separately to boys and girls because this is a small country and we are all related…..we are cousins …some females in the class are our aunties and certain topics can make them or us uncomfortable. In certain provinces, it is called “veitabuki”… males in their communities cannot approach or talk to the females, it is a taboo….but the school curriculum does not recognize that. My daughter is in Year 11. If I need her to know anything related to SRH, I will talk to the mom [my wife] for her to tell my daughter while for the boys, I will talk to them.”* [iTaukei male parent]
